# 基于倾向性评分匹配的单孔胸腔镜肺楔形切除术后免引流管策略安全性与临床获益分析

**DOI:** 10.3779/j.issn.1009-3419.2026.102.04

**Published:** 2026-02-20

**Authors:** Chutong LIN, Yingze NING, Shanwu MA, Jizheng TANG, Liang JIN, Wei HE, Huayu HE, Guangliang QIANG

**Affiliations:** 100191 北京，北京大学第三医院胸外科; Department of Thoracic Surgery, Peking University Third Hospital, Beijing 100191, China

**Keywords:** 肺肿瘤, 单孔胸腔镜, 肺楔形切除术, 胸腔闭式引流管, 倾向性评分匹配, 非劣效性研究, 贝叶斯分析, Lung neoplasms, Uniportal thoracoscopy, Wedge resection, Chest tube, Propensity score matching, Non-inferiority study, Bayesian analysis

## Abstract

**背景与目的:**

随着单孔胸腔镜技术在肺部小结节诊疗中的广泛应用，术后引流管管理的优化成为胸外科快速康复的关注点。本研究拟探讨单孔胸腔镜肺楔形切除术后免留置胸腔闭式引流管策略的安全性与非劣效性。

**方法:**

纳入2023年1月至2025年5月203例符合标准的患者，分为非引流组（*n*=53）与引流组（*n*=150），经倾向性评分匹配（propensity score matching, PSM）后获得41对均衡样本，采用非劣效性检验结合贝叶斯分析评估结局。

**结果:**

PSM后，非引流组在发热（19.51% *vs* 26.83%）、胸腔积液（24.39% *vs* 21.95%）发生率及额外加用镇痛药比例（12.20% *vs* 9.76%）上达非劣效标准（差值95%CI上限未超10%界值），中位术后住院日（2.00 *vs* 3.00 d, *P*<0.001）、术后首日视觉模拟评分（visual analogue scale, VAS）显著优于引流组（*P*=0.0495）且非劣效性成立；但二次干预（以二次置管为主）率（4.88% *vs* 0.00%）、影像学并发症（73.17% *vs* 65.85%）等指标非劣效性检验未成立（差值95%CI上限超10%界值）。贝叶斯分析显示，非引流组影像学相关并发症阳性率高于引流组的概率为77.18%，两组阳性率差异绝对值大于10.0%的概率为44.45%。

**结论:**

经严格筛选的低危患者术后免引流管策略可缩短住院时间、减轻疼痛，核心安全性指标达非劣效标准，但二次置管及影像学并发症相关非劣效性未达标，临床应用需加强术后监测以保障安全。

## Abstract (en)

**Background and objective:**

With the wide application of uniportal video-assisted thoracoscopy in the diagnosis and treatment of small pulmonary nodules, the optimization of postoperative chest tube management has become a focus of enhanced recovery after thoracic surgery. This study aimed to investigate the safety and non-inferiority of a no-drain strategy following uniportal video-assisted thoracoscopic wedge resection.

**Methods:**

A total of 203 eligible patients who underwent surgery between January 2023 and May 2025 were enrolled and allocated to the no-drain group (*n*=53) and the drain group (*n*=150). After propensity score matching (PSM), 41 well-balanced pairs were generated. Non-inferiority testing combined with Bayesian analysis was performed to assess postoperative outcomes.

**Results:**

After PSM, the no-drain group met the predefined non-inferiority criteria for the incidence of postoperative fever (19.51% *vs* 26.83%), pleural effusion (24.39% *vs* 21.95%), and the proportion of patients requiring additional analgesics (12.20% *vs* 9.76%), with the upper bound of the 95%CI for between-group differences not exceeding the 10% non-inferiority margin. The no-drain group demonstrated a significantly shorter median postoperative length of stay (2.00 *vs* 3.00 d, *P*<0.001) and lower visual analogue scale (VAS) scores on postoperative day 1 (*P*=0.0495), with non-inferiority confirmed. However, non-inferiority was not established for secondary intervention rates (primarily chest tube reinsertion, 4.88% *vs* 0.00%) or radiologic complications (73.17% *vs* 65.85%), as the upper limit of the 95%CI for the between-group differences exceeded the 10% margin. Bayesian analysis showed that the probability that the positive rate of imaging-related complications in the no-drain group was higher than that in the drain group was 77.18%, and the probability that the absolute value of the difference in positive rates between the two groups greater than 10.0% was 44.45%.

**Conclusion:**

In carefully selected low-risk patients, a no-drain strategy following uniportal thoracoscopic wedge resection may reduce postoperative pain and shorten hospital stay, while meeting non-inferiority criteria for key safety outcomes. However, non-inferiority was not demonstrated for secondary chest tube insertion or radiologic complications. Enhanced postoperative monitoring is therefore essential to ensure clinical safety.

肺癌是全球范围内发病率和死亡率最高的恶性肿瘤之一，严重威胁人类健康^[1-3]^。近年来，随着早期筛查技术的进步，越来越多的肺部小结节被检出，其中相当一部分需要手术切除以明确诊断或进行治疗^[4-6]^。单孔电视胸腔镜（uniportal video-assisted thoracoscopy, U-VATS）肺楔形切除术作为一种微创手术方式，因其创伤小、恢复快、美容效果好等优点，已成为处理肺部小结节的首选方法之一^[4,7]^。

胸腔引流管的放置在胸外科手术后有着悠久的历史，最早可追溯到1922年。自20世纪50年代以来，胸腔引流管一直是常规胸外科手术不可或缺的一部分。其主要功能是引流胸腔内的积气和积液，预防术后气胸，特别是张力性气胸的发生，并辅助医生判断是否需要再次手术干预^[8,9]^。然而，胸腔引流管本身也可能带来一系列并发症，如疼痛、不适、活动受限、感染风险增加以及住院时间延长等，这些因素均可能影响患者的术后恢复和生活质量。特别对于年轻患者，术后疼痛感可能更为强烈^[10]^。

鉴于传统胸腔引流管的潜在弊端，胸外科领域一直在探索优化引流策略，甚至尝试在特定情况下省略引流管。近年来，随着微创胸外科技术的发展及快速康复外科理念的推广，“选择性免留置胸腔闭式引流管”逐渐成为研究热点。其中，无管胸腔镜手术理念核心在于在严格术中漏气测试阴性前提下，减少甚至避免术后常规引流管留置，以减轻术后疼痛并促进快速康复^[11]^。

研究^[10]^表明，在VATS肺楔形切除术后省略胸腔引流管是一种安全的策略，并且能够加速患者康复。例如，一项系统评价和荟萃分析^[12]^指出，免引流管的U-VATS肺楔形切除术是安全的，且不会增加需要胸腔引流的气胸风险。另有研究^[4]^通过改进的引流策略，在U-VATS肺楔形切除术后实现了更短的术后康复时间、更少的疼痛和术后胸腔积液，并减轻了临床工作负担。此外，早期移除引流管甚至术中即刻移除引流管的策略，在处理自发性气胸的手术中也显示出其安全性和优势^[13]^。

尽管“免引流管”或“选择性留置引流管”的理念已逐渐被接受，但临床医生对于其安全性和普适性仍存在顾虑，尤其是在术后气胸等并发症的风险方面^[12]^。本研究旨在通过回顾性队列研究，并结合倾向性评分匹配（propensity score matching, PSM）和贝叶斯分析等统计方法，全面评估U-VATS肺楔形切除术后免引流管策略的安全性与有效性。本研究希望明确免引流管策略在疼痛控制、住院日、并发症发生率和二次干预率等方面的表现，并深入探讨其与传统引流策略之间的差异及其临床意义。本研究将为临床实践提供更精准的循证医学证据，以期优化U-VATS肺楔形切除术后的引流管管理策略，进一步提升患者的快速康复效果。

## 1 资料与方法

### 1.1 研究人群

本研究为回顾性队列研究，研究对象来源于来自本研究中心2023年1月至2025年5月收治的接受U-VATS肺楔形切除术的外周型早期肺癌患者，根据术后是否留置胸腔闭式引流管，将患者分为非引流组（不留置胸腔闭式引流管）与引流组（留置胸腔闭式引流管）。本研究已经过北京大学第三医院伦理委员会审批，伦理批件编号为（2025）医伦第（728-01）号。

纳入本研究的患者需同时满足以下条件：（1）接受单侧U-VATS肺楔形切除；（2）肺部病灶为周围型肺结节，距离脏层胸膜≤2 cm且直径<2 cm，临床高度怀疑恶性肿瘤；（3）年龄在18-75岁之间，具备完全的临床评估及手术耐受基础；（4）肺功能检查提示第一秒用力呼气容积（forced expiratory volume in one second, FEV_1_）≥预计值的60%；（5）病历资料完整，可满足研究数据采集需求。

存在以下任一情况的患者均予以排除：（1）既往疾病方面，中重度慢性阻塞性肺疾病（chronic obstructive pulmonary disease, COPD）或限制性肺疾病[支气管扩张剂使用后FEV_1_<预计值80%且FEV_1_/用力肺活量（FEV_1_/forced vital capacity, FEV_1_/FVC）<70%]、有既往胸部手术史、合并充血性心力衰竭、出血倾向、肝硬化、慢性肾病等严重基础疾病或需长期使用抗凝剂、类固醇药物，或存在需静脉使用抗生素的活动性感染、存在出血风险增加因素或凝血功能障碍、长期存在胸部慢性疼痛或因各种原因需长期口服止疼药；（2）手术相关方面，手术过程中中转开胸、除肺楔形切除±淋巴结采样/清扫及粘连松解外接受了其他额外手术方式或麻醉方式干预、术中存在严重胸膜粘连且分解粘连时间>30 min，术中发现肺组织存在漏气者；（3）特殊情况方面，处于妊娠或哺乳期的女性、存在认知功能障碍无法配合临床评估，或病历资料记录不全、关键信息缺失影响数据完整性。

本研究通过标准化的数据采集表系统提取两组患者的基线信息，所有数据采集均由经过统一培训的研究人员完成，确保数据的准确性和一致性。

### 1.2 手术流程

U-VATS手术遵循标准流程^[7]^。患者健侧卧位，全麻后双腔气管插管、健侧单肺通气。于腋前线第4或5肋间作3-4 cm切口。探查胸腔后，保证切缘距病灶≥2 cm（或≥1倍最大直径），使用切割闭合器完成肺楔形切除。冲洗胸腔，探查有无出血及漏气，仅无肺漏气的患者纳入本研究。关胸前，根据需要留置纤丝类、膜类、胶类等生物材料。一般情况下，对于淋巴结清扫部位使用纤丝类或胶类止血材料，对于肺切缘使用膜类材料预防漏气。

后续，引流组于切口背侧放置1根24 Fr的胸腔闭式引流管，导管末端置于胸顶部，常规鼓肺、关胸。非引流组在关胸前行漏气测试，具体操作及判定标准如下：（1）前置准备：于切口背侧留置1根24 Fr胸腔闭式引流管，于胸壁肌肉层行间断缝合，引流管周围缝线暂不打结，预留关胸通道。此时经切口置入胸腔镜，直视下嘱麻醉医师行术侧单肺正压通气，固定通气参数：气道峰压25 cmH₂O、呼气末正压5 cmH₂O，维持该参数使术侧肺完全复张后，撤出胸腔镜。（2）测试系统连接与通气设置：将留置的胸腔闭式引流管末端连接闭式引流瓶，命助手捏住胸壁切口以避免伤口漏气，嘱麻醉医师行术侧单肺正压通气，通气参数同上。（3）观察时长与漏气判定标准：维持上述正压通气参数，持续观察30 s，全程记录引流瓶内气泡溢出情况，判定标准如下：漏气试验阳性（失败）：30 s观察期内，引流瓶水下管端持续存在气泡溢出，判定为存在肺组织漏气。该病例需常规留置胸腔闭式引流管，不再纳入本研究；漏气试验阴性（通过）：30 s观察期内，引流瓶水下管端无气泡溢出，或仅在通气初始肺进一步复张阶段出现一过性气泡、后续15 s内完全无气泡溢出，判定为无肺组织漏气，试验通过。

试验通过后关胸操作：试验通过后，维持麻醉机正压通气状态，将闭式引流瓶连接负压吸引装置，设置负压值为-10--15 cmH₂O，持续抽吸胸膜腔内残余气体；同时在负压吸引状态下，快速将胸腔套管从胸腔内拔出，套管完全退出后，即刻收紧预留的间断缝线并打结固定，闭合胸壁创口，确认创口无渗气、渗液。

手术的镇痛方案包括肋间神经阻滞（intercostal nerve block, ICNB）、患者自控镇痛泵（patient-controlled analgesia, PCA）、单次神经阻滞、单次静脉镇痛，麻醉医生根据需要及患者意愿，为患者选择不同的镇痛方式。术后当晚所有患者例行接受坐位床旁胸片检查一次，由医生判断非引流组是否需要额外留置引流管以及引流组的术后拔管条件。所有患者均于拔管后3周门诊复查拆线，并复查胸片。

### 1.3 结局指标

本研究的主要研究结局指标为术后二次干预率及并发症发生率。

其中，术后并发症包括影像学相关并发症与发热，影像学相关并发症依据出院前最严重的胸片检查结果判定，具体涵盖气胸、胸腔积液（胸片显示手术部位肋膈角变钝）、迟发性胸腔积液（术后2周及以上拆线时复查胸片提示手术部位肋膈角变钝）、皮下气肿（胸片显示从手术切口延伸的皮下气体潴留现象）。发热定义为住院期间体温超过37.5 ^o^C。

二次干预及预后不良相关指标包括因气胸、胸腔积液、皮下气肿或其他原因需重新置入胸管、行胸腔穿刺术（含迟发性胸腔积液处理）的再次干预情况，术后30 d内是否因各类原因需再次手术、是否再次入院，以及住院期间或术后30 d内的死亡情况。

次要研究结局以疼痛相关指标为核心，包括术后当日、术后首日的视觉模拟评分（visual analogue scale, VAS）（0-10分，若单日多次报告则取最高值，分数越高，疼痛越剧烈）、是否在常规镇痛方案基础上追加其他镇痛药物和术后住院日。

### 1.4 统计学方法

本研究采用Python（3.12.7）完成所有数据的统计分析，主要借助scipy.stats模块（SciPy 1.14.0）实现各类统计学检验，结合pandas（2.2.3）完成数据清洗与整理，numpy（1.26.4）辅助数值计算。检验水准设定为α=0.05，*P*<0.05为差异具有统计学意义。研究数据先进行正态性检验与方差齐性检验，符合正态分布且方差齐的连续变量以均数±标准差（Mean±SD）表示，组间的比较采用独立样本*t*检验；不符合正态分布的连续变量以中位数（四分位数间距）[M(Q1, Q3)]表示，组间比较采用非参数检验中的*Mann-Whitney U*检验。计数资料与二元变量以例数（构成比）[*n*(%)]表示，组间率的比较采用χ²检验，若理论频数<5则采用*Fisher*确切概率法。各项结局指标同步行优效性检验及非劣效性检验，其中非劣效性界值根据临床经验设置为10%。

本研究采用PSM平衡组间混杂因素，选取患者性别、手术时年龄、淋巴结清扫、术中止血材料使用、主要病灶部位、患者吸烟史为协变量，以患者住院号为索引，通过PSMPY包（0.3.13）构建逻辑回归模型计算倾向性评分，采用无放回k近邻匹配（匹配依据为倾向性评分logit值，卡钳值0.30），并通过绘制倾向性评分分布密度图、标准化均值差效应量图验证匹配平衡性，用于后续分析。

本研究针对PSM后的数据，采用贝叶斯逻辑回归分析胸引管留置与影像学并发症发生风险的关联，以弱信息先验（截距项和回归系数均服从均值为0、标准差为10的正态分布）构建模型，通过无回绕采样算法（No-U-Turn Sampler, NUTS）这一马尔可夫链蒙特卡洛（Markov Chain Monte Carlo, MCMC）采样算法进行2000次有效采样（预烧期1000次，目标接受率0.95），从MCMC采样得到的后验分布中提取截距项和回归系数样本，计算两组结局阳性率的后验样本，以及组间阳性率差异和相对风险的后验样本，采用ArviZ库（0.22.0）获取各指标的后验统计量，包括均值、标准差、95%最高密度区间（highest density interval, HDI），同时计算非引流组并发症率低于引流组的概率、组间差异绝对值>10%的概率及相对风险>1.1的概率，并借助Matplotlib库（3.9.2），通过核密度估计（kernel density estimation, KDE）曲线可视化两组并发症阳性率的后验分布特征，量化评估胸引管留置对影像学并发症发生风险的影响及结果的临床意义。

## 2 结果

### 2.1 研究人群特征

本研究共纳入2023年1月-2025年5月于北京大学第三医院接受U-VATS肺楔形切除术的203例外周型早期肺癌患者，依据术后是否留置胸腔闭式引流管分为引流组（*n*=150）与非引流组（*n*=53）。两组患者基线资料对比显示，身高、体重、体质指数（body mass index, BMI）等一般体格指标，性别构成、吸烟史、术前FEV_1_实测值、FEV_1_占预计值百分比、FEV_1_/FVC比值等肺功能指标，以及肺部主要病灶大小比较，差异均无统计学意义（均*P*>0.05）。

两组间差异方面，非引流组患者年龄整体低于引流组（*P*=0.0014）；引流组行淋巴结采样的比例（77.3%）高于非引流组（47.2%）（*P*=0.0001）；引流组右肺上叶病灶占比（32.0%）高于非引流组（13.2%），而非引流组左肺上叶、右肺中叶病灶占比相对更高（*P*=0.0165）。术中止血材料使用方面，引流组纤丝类材料使用占比（68.7%）高于非引流组（41.5%）（*P*=0.0005），非引流组膜类材料使用占比（98.1%）则显著高于引流组（62.7%）（*P*<0.0001）（表1）。

**表1:** 研究人群特征

Item	Drain group (*n*=150)	No-drain group (*n*=53)	*P*
Age at surgery (yr)	55.00 (43.00-63.75)	51.00 (38.00-55.00)	0.0014
Height (cm)	163.00 (158.00-170.00)	163.00 (160.00-169.00)	0.9761
Weight (kg)	65.00 (58.00-72.00)	63.00 (55.00-73.00)	0.3381
BMI (kg/m^2^)	24.43±3.28	23.68±2.94	0.1426
Preoperative pulmonary function - FEV_1_ (L)	2.61 (2.23-3.03)	2.61 (2.31-3.21)	0.4519
Preoperative pulmonary function - FEV_1_ (%)	98.00 (89.00-106.95)	98.00 (90.00-106.00)	0.9609
Preoperative pulmonary function - FEV_1_/FVC	79.11 (77.00-83.00)	79.48 (76.18-84.35)	0.5006
Nodule characteristics - Maximum lesion size (mm)	10.00 (8.00-12.00)	9.00 (7.00-12.00)	0.3618
Gender			0.6860
Female	106 (70.7%)	39 (73.6%)	
Male	44 (29.3%)	14 (26.4%)	
Smoking history			0.6954
Never smoked	133 (88.7%)	47 (88.7%)	
Quit smoking	11 (7.3%)	5 (9.4%)	
Current smoker	6 (4.0%)	1 (1.9%)	
Operation time (min)	54.00 (45.00-66.00)	50.00 (41.00-60.00)	0.1384
Blood loss volume (mL)	10.00 (10.00-10.00)	10.00 (5.00-10.00)	0.5645
Lymph node sampling			0.0001
Yes	116 (77.3%)	25 (47.2%)	
No	34 (22.7%)	28 (52.8%)	
Primary lesion location			0.0165
LUL	42 (28.0%)	19 (35.8%)	
LLL	26 (17.3%)	11 (20.8%)	
RUL	48 (32.0%)	7 (13.2%)	
RML	7 (4.7%)	8 (15.1%)	
RLL	27 (18.0%)	8 (15.1%)	
Postoperative pathological diagnosis			0.9308
Primary lung cancer	126 (84.0%)	45 (84.9%)	
Benign lesion	22 (14.7%)	7 (13.2%)	
Metastatic tumor	2 (1.3%)	1 (1.9%)	
Intraoperative hemostatic material application			
Fibrillar type	103 (68.7%)	22 (41.5%)	0.0005
Membranous type	94 (62.7%)	52 (98.1%)	<0.0001
Gel type	145 (96.7%)	53 (100.0%)	0.4063
Analgesic regimen			
PCA	124 (82.7%)	47 (88.7%)	0.3018
ICNB	37 (24.7%)	9 (17.0%)	0.2506
Single intravenous analgesia	122 (81.3%)	41 (77.4%)	0.5317
Single nerve block	38 (25.3%)	10 (18.9%)	0.3410

BMI: body mass index; FEV_1_: forced expiratory volume in one second; FVC: forced vital capacity; LUL: left upper lobe; LLL: left lower lobe; RUL: right upper lobe; RML: right middle lobe; RLL: right lower lobe; PCA: patient-controlled analgesia; ICNB: intercostal nerve block.

### 2.2 PSM前两组患者结局比较结果

本研究对引流组（*n*=150）与非引流组（*n*=53）的各项结局指标同步行优效性检验及非劣效性检验。优效性检验结果显示，非引流组术后住院日、术后首日VAS评分均显著低于引流组，非引流组二次干预显著高于引流组（*P*=0.023），其余指标组间比较均无统计学差异（*P*均>0.05）。

非劣效性检验结果显示，共6项指标检验成立，包括发热（16.98% *vs* 18.67%，差值95%CI: -∞-8.28%）、胸腔积液（24.53% *vs* 37.33%，差值95%CI: -∞--1.11%）、加用止疼药（11.32% *vs* 13.33%，差值95%CI: -∞-6.48%），以及术后住院日、手术日VAS评分、术后首日VAS评分；二次干预、围手术期并发症发生率、整体影像学并发症、气胸、迟发性胸腔积液、皮下气肿这些指标，组间差值95%CI上限均超10%界值，非劣效性检验均不成立（表2）。

**表2:** PSM前两组患者结局比较结果

Variable	No-drain group (*n*=53)	Drain group (*n*=150)	P value for superiority test	Non-inferiority test		
				Group difference (95%CI)	Non-inferiority margin	Non-inferiority result
Secondary intervention	5.66% (3/53)	0.00% (0/150)	0.0230	-∞-10.88%	10%	Not established
Perioperative complication rate	79.25% (42/53)	77.33% (116/150)	0.7730	-∞-12.66%	10%	Not established
Fever	16.98% (9/53)	18.67% (28/150)	0.7847	-∞-8.28%	10%	Established
Imaging complications	77.36% (41/53)	69.33% (104/150)	0.2663	-∞-19.33%	10%	Not established
Pneumothorax	15.09% (8/53)	8.00% (12/150)	0.1363	-∞-15.97%	10%	Not established
Pleural effusion	24.53% (13/53)	37.33% (56/150)	0.0907	-∞--1.11%	10%	Established
Delayed pleural effusion	16.98% (9/53)	10.00% (15/150)	0.1760	-∞-16.37%	10%	Not established
Subcutaneous emphysema	50.94% (27/53)	38.67% (58/150)	0.1194	-∞-25.33%	10%	Not established
Additional analgesic administration	11.32% (6/53)	13.33% (20/150)	0.7063	-∞-6.48%	10%	Established
Postoperative hospital stay (d)	2.00 (2.00-2.00)	3.00 (2.00-3.00)	<0.0001	-∞--1.00	0.30	Established
VAS score_Operative day	3.00 (2.00-3.00)	3.00 (2.00-3.00)	0.9681	-∞-0.00	0.30	Established
VAS score_ Postoperative d1	2.00 (2.00-3.00)	3.00 (2.00-3.00)	0.0030	-∞-0.00	0.30	Established

PSM: propensity score matching; VAS: visual analogue scale.

### 2.3 PSM后两组患者基线特征的平衡性分析

按照前文所述方法进行PSM后，共匹配41对患者，两组结果的组间差异得到较好的平衡，基线特征不再具有统计学差异（表3）。

**表3:** PSM后研究人群特征

Item	Drain group (*n*=41)	No-drain group (*n*=41)	*P*
Age at surgery (yr)	47.00 (37.00-61.00)	52.00 (38.00-55.00)	0.7034
Height (cm)	162.00 (160.00-170.00)	163.00 (160.00-170.00)	0.6758
Weight (kg)	62.00 (54.00-72.00)	63.00 (55.00-73.00)	0.9482
BMI (kg/m^2^)	23.49±3.87	23.60±2.96	0.8770
Preoperative pulmonary function - FEV_1_ (L)	2.61 (2.24-3.23)	2.61 (2.31-3.16)	0.8021
Preoperative pulmonary function - FEV_1_ (%)	98.00 (87.00-104.50)	97.00 (90.00-106.00)	0.8346
Preoperative pulmonary function - FEV_1_/FVC	79.29 (78.00-84.00)	79.22 (76.18-84.00)	0.3755
Nodule characteristics - Maximum lesion size (mm)	10.00 (8.00-12.00)	9.00 (7.00-12.00)	0.1648
Gender			0.8058
Female	30 (73.2%)	29 (70.7%)	
Male	11 (26.8%)	12 (29.3%)	
Smoking history			0.0944
Never smoked	38 (92.7%)	36 (87.8%)	
Quit smoking	1 (2.4%)	5 (12.2%)	
Current smoker	2 (4.9%)	0 (0.0%)	
Operation time (min)	50.00 (44.00-61.00)	50.00 (41.00-57.00)	0.3558
Blood loss volume (mL)	10.00 (10.00-10.00)	10.00 (5.00-10.00)	0.5386
Lymph node sampling			0.6572
Yes	24 (58.5%)	21 (51.2%)	
No	17 (41.5%)	20 (48.8%)	
Primary lesion location			0.4862
LUL	13 (31.7%)	16 (39.0%)	
LLL	9 (22.0%)	8 (19.5%)	
RUL	7 (17.1%)	5 (12.2%)	
RML	3 (7.3%)	7 (17.1%)	
RLL	9 (22.0%)	5 (12.2%)	
Postoperative pathological diagnosis			0.5678
Primary lung cancer	32 (78.0%)	35 (85.4%)	
Benign lesion	0 (0.0%)	0 (0.0%)	
Metastatic tumor	9 (22.0%)	6 (14.6%)	
Intraoperative hemostatic material application			
Fibrillar type	18 (43.9%)	19 (46.3%)	0.8244
Membranous type	41 (100.0%)	40 (97.6%)	0.9999
Gel type	38 (92.7%)	41 (100.0%)	0.2394
Analgesic regimen			
PCA	36 (87.8%)	37 (90.2%)	0.9999
ICNB	10 (24.4%)	9 (22.0%)	0.7935
Single intravenous analgesia	34 (82.9%)	31 (75.6%)	0.4138
Single nerve block	11 (26.8%)	6 (14.6%)	0.1732

### 2.4 PSM后两组患者结局比较结果

PSM后，两组患者结局比较见表4。优效性检验结果显示，非引流组术后住院日、术后首日VAS评分仍显著低于引流组；二次干预两组间比较结果发生变化，匹配后二者无统计学差异（*P*=0.4741）。其余各项指标组间优效性检验同前。

**表4:** PSM后两组患者结局比较结果

Variable	No-drain group (*n*=41)	Drain group (*n*=41)	P value for superiority test	Non-inferiority test		
				Group difference (95%CI)	Non-inferiority margin	Non-inferiorit result
Secondary intervention	4.88% (2/41)	0.00% (0/41)	0.4741	-∞-10.88%	10%	Not established
Perioperative complication rate	75.61% (31/41)	75.61% (31/41)	0.9999	-∞-15.60%	10%	Not established
Fever	19.51% (8/41)	26.83% (11/41)	0.4323	-∞-8.28%	10%	Established
Imaging complications	73.17% (30/41)	65.85% (27/41)	0.4717	-∞-23.99%	10%	Not established
Pneumothorax	17.07% (7/41)	9.76% (4/41)	0.3310	-∞-15.97%	10%	Not established
Pleural effusion	24.39% (10/41)	21.95% (9/41)	0.7935	-∞--1.11%	10%	Established
Delayed pleural effusion	17.07% (7/41)	14.63% (6/41)	0.7624	-∞-16.37%	10%	Not established
Subcutaneous emphysema	46.34% (19/41)	34.15% (14/41)	0.2602	-∞-25.33%	10%	Not established
Additional analgesic administration	12.20% (5/41)	9.76% (4/41)	0.9999	-∞-6.48%	10%	Established
Postoperative hospital stay (d)	2.00 (2.00-2.00)	3.00 (2.00-3.00)	<0.0001	-∞-1.00	0.30	Established
VAS score_Operative day	3.00 (2.00-3.00)	3.00 (2.00-3.00)	0.9999	-∞-0.00	0.30	Established
VAS score_ Postoperative d1	2.00 (2.00-3.00)	3.00 (2.00-3.00)	0.0495	-∞-0.00	0.30	Established

非劣效性检验结果显示，共6项指标检验成立，包括发热（19.51% *vs* 26.83%，差值95%CI: -∞-8.28%）、胸腔积液（24.39% *vs* 21.95%，差值95%CI: -∞--1.11%）、加用止疼药（12.20% *vs* 9.76%，差值95%CI: -∞-6.48%），以及术后住院日、手术日VAS评分、术后首日VAS评分；二次干预、围手术期并发症发生率、影像学并发症、气胸、迟发性胸腔积液、皮下气肿这些指标，组间差值95%CI上限均超10%界值，非劣效性检验均不成立，结果同前。

### 2.5 针对影像学并发症的贝叶斯统计分析

在PSM前后，影像学并发症在两组之间的有效性检验均为无意义，非劣效性检验均不成立。为进一步探索是否留置引流管对影像学并发症的影响效应，本研究进行了贝叶斯统计分析。

图1展示了贝叶斯分析中非引流组与引流组影像学并发症发生概率的后验分布特征：从分布形态看，非引流组的后验分布峰值更靠近较高概率区间，而引流组的分布峰值相对更集中于中等概率区间，提示非引流组影像学并发症发生概率的后验估计值整体高于引流组。

**图1:**
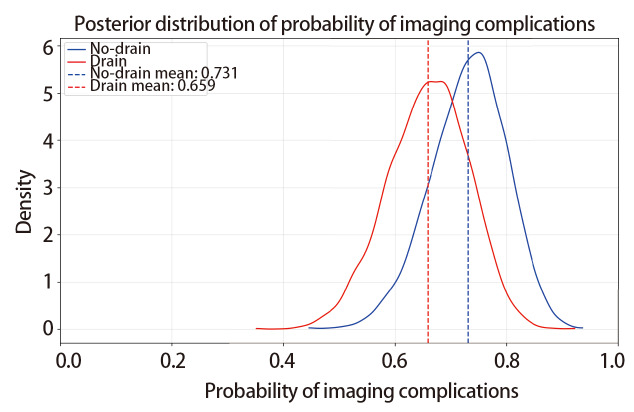
非引流组与引流组患者影像学并发症发生概率后验分布的核密度估计曲线图。图中蓝色实线代表非引流组，红色实线代表引流组；蓝色虚线为非引流组影像学并发症发生概率的后验均值（0.731），红色虚线为引流组影像学并发症发生概率的后验均值（0.659）。横轴为影像学并发症的发生概率，纵轴为核密度估计对应的密度值。

图2展示了非引流组与引流组影像学并发症发生率差值的后验分布特征：该差值的后验均值为0.072，95% HDI覆盖-0.130-0.270，提示两组并发症发生率的差异存在一定不确定性；同时，该差值的后验分布中有55.9%的样本落在临床无意义区间（region of practical equivalence, ROPE）-0.1-0.1内，结合分布形态与区间范围可知，两组影像学并发症发生率的差异尚未达到明确的临床显著水平。

**图2:**
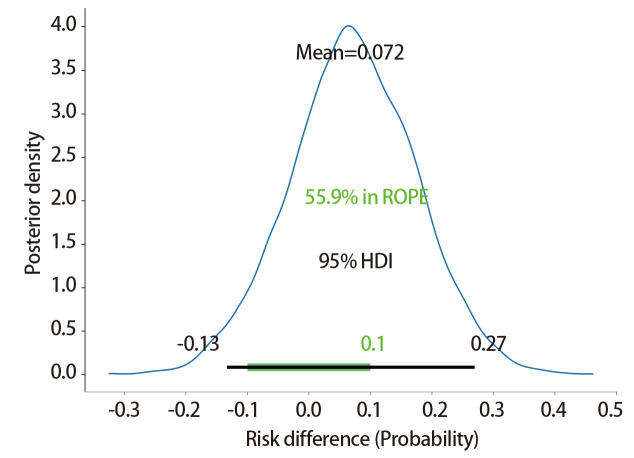
非引流组与引流组患者影像学并发症发生率差值的后验分布及95% HDI图。图中蓝色曲线为两组影像学并发症发生率差值的后验分布核密度曲线，差值计算方式为非引流组发生率减去引流组发生率；后验分布均值为0.072，95% HDI为-0.130-0.270（黑色横线标注）；临床ROPE为-0.1-0.1（绿色线段标注），后验分布中落在ROPE区间的比例为55.9%。横轴为两组影像学并发症发生率的差值，纵轴为核密度估计对应的密度值。

最终通过计算得知，非引流组影像学相关并发症阳性率高于引流组的概率：77.18%，两组阳性率差异绝对值>10.0%的概率：44.45%，相对风险>1.1的概率：53.70%。

## 3 讨论

本研究为单中心回顾性非劣效性队列研究，纳入203例U-VATS肺楔形切除术患者（非引流组53例、引流组150例），经PSM后获得41对均衡样本。核心结果显示，无论PSM前后，非引流组术后住院日、术后首日VAS疼痛评分均显著低于引流组，且发热、胸腔积液发生率及额外加用镇痛药比例的非劣效性检验成立；PSM前非引流组二次干预率更高，PSM后该差异消失，但非劣效性检验始终不成立。针对总体影像学并发症，虽两组非劣效性检验均不成立，但贝叶斯分析显示，55.9%的后验样本落在ROPE内，提示差异无明确临床意义。

在引流管留置必要性的争议方面，本研究发现选择性留置引流管或免引流管策略在改善患者舒适度方面具有优势，这与多项研究的结论相符。例如，有研究^[12]^表明，VATS手术后避免常规放置胸腔引流管是安全的，并且能够促进患者康复。U-VATS手术在减轻术后疼痛、缩短住院时间及引流管留置时间方面显示出相较于传统VATS手术的优势^[14]^。此外，一项针对肺楔形切除术后改进引流策略的研究^[4]^也指出，改进引流策略能有效减轻术后疼痛，缩短住院时间。另一项研究^[15]^发现，与常规20-Fr胸腔引流管相比，使用12-Fr猪尾导管作为胸腔引流管能显著降低伤口并发症发生率和咳嗽时疼痛评分。这些研究共同支持了本研究关于非引流组疼痛减轻和住院日缩短的发现，并强调了在特定患者群体中采用免引流策略的可行性。然而，传统观点认为胸腔引流管在胸外科手术后是必要的，旨在引流胸腔内液体和气体，预防气胸^[8]^。部分研究^[16]^也强调了引流管在监测术后出血和气漏方面的作用。因此，免引流管策略并非适用于所有患者，其安全应用关键在于严格把控适应证与禁忌证、精准筛选人群，以此发挥优势并规避并发症，这也是本研究制定排除标准的依据。结合术中标准化流程与临床证据，胸腔粘连严重、分离后胸膜广泛损伤，渗血渗液风险高者，不宜免管；肺实质差、愈合能力弱，迟发性漏气风险高者亦不宜免管。另外，术中出血多、止血不佳或凝血异常，需引流监测者，需要引流管作为基本检测手段。所以，术前全面评估、术中及时识别风险是制定个体化引流方案的核心。

本研究对并发症的特殊视角也值得深入探讨。本研究中非引流组术后气胸、迟发性胸腔积液、皮下气肿等指标的非劣效性检验均未成立，提示无管技术仍存在潜在安全风险。针对非引流组3例二次置管病例，我们补充了其临床特征的系统分析，3例患者均为年轻患者、左肺上叶楔形切除、术前肺功能良好、术中出血少且均通过标准化漏气测试，其中2例未行淋巴结采样。病例1和病例3均于手术日晚间床旁胸片发现了术后出血，后续证实均为伤口出血，与手术切除部位——左肺上叶关联不大，因此对于无管患者，需更加注意关胸前的伤口止血以及术后监测。病例2术后胸片未见明显积液，但3周复查胸片发现胸腔内中等量胸腔积液。此类病例术后即使留置引流，无法避免迟发性积液的发生，因此不存在术后无管禁忌。有研究^[17]^发现，系统性淋巴结清扫会增加术后渗出风险，因而影响到术后胸腔积液的发生率。注意到此例迟发性积液病例同样发生于左肺上叶楔形切除，但目前的临床研究和专家观点并未提供确凿证据表明左肺上叶楔形切除术更容易引起术后胸腔积液。积液的发生是一个多因素作用的结果，单独的一例患者也难以证实楔形切除部位与术后积液的相关性。本研究中PSM前两组淋巴结清扫方式存在分布差异，非引流组纵隔淋巴结采样率更低，所以这可以解释围手术期引流组胸腔积液发生率稍低于非引流组。然而，迟发性胸腔积液的发生率则是非引流组更高，且未达非劣效，并因此产生了二次置管病例，因而留置引流管对胸腔积液的复杂影响仍需更进一步探索。此外，二次置管虽可解决患者术后安全康复的相关问题，但其给患者带来的焦虑情绪与躯体疼痛，仍值得每一位外科医生高度重视与审慎反思。

本研究两组患者气胸发生率较高，或与并发症定义、评估时机、研究人群和其他研究不同有关。有研究^[13]^表明，自发性气胸VATS术后不留置胸腔引流管不增加气胸风险；荟萃分析^[12]^也证实，VATS肺切除术后不常规置管安全可行，不会提升再次引流风险。但也有研究^[18]^认为，部分情况下不留置引流管会增加术后气胸风险，差异或与患者选择、手术类型、术后监测有关。本研究经PSM严格限定手术类型与患者特征，结果更具同质性；而既往强调引流必要性的研究可能纳入了混合术式（如肺段切除、袖式切除）或未严格排除高危患者（如COPD、肺气肿），这可能导致了影像学异常率的差异^[19]^。

在本研究中，非引流组的影像学并发症整体水平相对更高。贝叶斯分析显示，非引流组影像学并发症阳性率高于引流组的概率为77.18%，该结果仅提示免引流管策略存在影像学异常风险略高的趋势，并非“风险显著增高”的确定性结论。结合后验分布：两组并发症率差值的HDI为-0.130-0.270，同时覆盖0与10%非劣效界值，提示组间差异存在较大统计学不确定性；且55.9%的后验样本落在（-0.1-0.1）的ROPE内，差异绝对值超10%界值的概率仅44.45%，说明该风险差异尚未达到明确的临床意义，需避免对结果的片面误读。

本研究联合贝叶斯分析与传统非劣效性检验，实现了临床决策上的方法互补^[20]^：非劣效性检验基于预设界值给出二元化判断，明确免引流管策略在影像学并发症、二次干预率等终点的非劣效性暂未成立；贝叶斯分析则突破频率学统计仅关注*P*值的局限，量化了组间差异的不确定性，补充了非劣效检验未覆盖的决策信息——尽管非劣效性未成立，但潜在风险升高未达临床有意义程度，为低危患者个体化选择引流策略提供了精细化循证参考。

在统计方法的优势与创新方面，本研究结合了PSM与贝叶斯分析，这为回顾性队列研究提供了一种强大的统计学方法。PSM有助于平衡混杂因素，而贝叶斯方法能够量化不确定性，并结合ROPE评估差异的临床意义，从而补充了传统非劣效性检验的结果。这种结合方法能够更全面、更细致地评估两种引流策略的安全性与有效性，为临床决策提供了更可靠的证据。

本研究仍存在一些局限性。例如，单中心回顾性设计、样本量有限（非引流组尤为明显）、缺乏长期随访等局限性，未来尚需通过多中心前瞻性队列研究或随机对照试验进一步验证相关结论。另外，本研究基于人群，分析了无管方案的安全性和有效性，但临床仍需个性化方案。在未来，需进一步研究影响并发症发生的因素，并探讨更适合无管方案的患者的个性化决策方法。

综上所述，本研究围绕U-VATS肺楔形切除术后免引流管策略的安全性展开探究，所得结果既与现有文献结论相契合，也契合近年微创胸外科“选择性免引流”的临床趋势，均证实该策略在减轻患者术后疼痛、缩短住院日方面的优势。同时，本研究通过PSM有效平衡基线混杂因素，结合严谨的影像学并发症定义与先进的贝叶斯统计分析，不仅揭示出与既往研究的差异或源于研究方法学和患者选择的细节，还量化了研究结果的不确定性，弥补了传统频率学统计仅关注*P*值的局限，为胸外科术后引流管管理的个体化策略提供了重要的循证支持。在临床实践中，对于术前肺功能良好、术中无漏气的外周型小病灶患者，可优先考虑采用免引流策略，同时需加强术后胸片监测，及时处理迟发性胸腔积液等情况，以实现术后“快速康复”与“安全可控”的平衡。
